# Experience of Gastrostomy Using a Quality Care Framework: The Example of Rett Syndrome

**DOI:** 10.1097/MD.0000000000000328

**Published:** 2014-12-02

**Authors:** Jenny Downs, Kingsley Wong, Madhur Ravikumara, Carolyn Ellaway, Elizabeth J. Elliott, John Christodoulou, Peter Jacoby, Helen Leonard

**Affiliations:** From the Telethon Kids Institute (JD, KW, PJ, HL), The University of Western Australia; School of Physiotherapy and Exercise Science (JD), Curtin University, Perth, Australia; Department of Gastroenterology (MR), Princess Margaret Hospital, Perth, Australia; Discipline of Genetic Medicine (CE, JC); Discipline of Paediatrics and Child Health (CE, EJE, JC), The University of Sydney, The Children's Hospital at Westmead; and The Sydney Children's Hospitals Network (Westmead) (CE, EJE, JC), Sydney, Australia.

## Abstract

Rett syndrome is one of many severe neurodevelopmental disorders with feeding difficulties. In this study, associations between feeding difficulties, age*, MECP2* genotype, and utilization of gastrostomy were investigated. Weight change and family satisfaction following gastrostomy were explored.

Data from the longitudinal Australian Rett Syndrome Database whose parents provided data in the 2011 family questionnaire (n = 229) were interrogated. We used logistic regression to model relationships between feeding difficulties, age group, and genotype. Content analysis was used to analyze data on satisfaction following gastrostomy.

In those who had never had gastrostomy and who fed orally (n = 166/229), parents of girls <7 years were more concerned about food intake compared with their adult peers (odds ratio [OR] 4.26; 95% confidence interval [CI] 1.29, 14.10). Those with a p.Arg168^∗^ mutation were often perceived as eating poorly with nearly a 6-fold increased odds of choking compared to the p.Arg133Cys mutation (OR 5.88; 95% CI 1.27, 27.24). Coughing, choking, or gagging during meals was associated with increased likelihood of later gastrostomy. Sixty-six females (28.8%) had a gastrostomy, and in those, large *MECP2* deletions and p.Arg168^∗^ mutations were common. Weight-for-age z-scores increased by 0.86 (95% CI 0.41, 1.31) approximately 2 years after surgery. Families were satisfied with gastrostomy and felt less anxious about the care of their child.

Mutation type provided some explanation for feeding difficulties. Gastrostomy assisted the management of feeding difficulties and poor weight gain, and was acceptable to families. Our findings are likely applicable to the broader community of children with severe disability.

## INTRODUCTION

Rett syndrome is a severe neurodevelopmental disorder, occurring mainly in females and usually associated with a mutation of the methyl-CpG-binding protein 2 (*MECP2*) gene.^1^ It occurs rarely^2^ with a wide phenotypic spectrum in part explained by the specific *MECP2* mutation.^3^ Following apparently normal early development, there is loss of communication and hand function with the development of intense midline hand stereotypies. Comorbidities include breathing dysfunction, scoliosis,^4^ epilepsy,^5^ and gastrointestinal disorders.^6^

Poor growth is common in Rett syndrome.^7^ The *MECP2* mutation likely has a role, but other contributing factors include poor muscle tone and oromotor incoordination, which can cause feeding difficulties.^8^ Gastrointestinal dysmotility may lead to gastroesophageal reflux disorder, delayed gastric emptying, and/or constipation.^9^ Many have disturbed breathing patterns with episodic hyperventilation, breath holding, aerophagia, and abdominal distension causing discomfort and reduced oral intake. While some appear to have a good appetite,^10^ many parents have concerns about their daughter's feeding pattern and intake.

Rett syndrome is one of many genetic disorders associated with severe disability in which feeding difficulties and poor growth occur.^11^ Conservative management is trialed initially,^9,10^ but with persistent feeding difficulties and poor weight gain, gastrostomy may be recommended.^10^ There are many advantages to gastrostomy for children with a severe developmental disability, including improved nutrition and growth, as well as reduced carer anxiety. A Spanish study involving 26 parents of children with severe developmental disability reported high satisfaction levels in relation to their child's health, daily care routines, and family dynamics following gastrostomy.^12^ Despite these advantages,^13^ parents are often slow to accept a gastrostomy as the best management for their child.^14^ In Rett syndrome, gastrostomy has been associated with improved growth^15^ but there has been no comprehensive investigation of outcomes using a quality of care framework.^16^

The Australian Rett Syndrome Database (ARSD) is unique worldwide in collecting genetic and phenotype data that is both population based and longitudinal.^17^ This dataset allows us to look back at factors that preceded gastrostomy and assess outcomes following surgery such as growth. Our Rett syndrome data could also provide insights into issues encountered by children with other severe disabilities. Using data collected in the ARSD, we investigated feeding difficulties in Rett syndrome and their relationships to age and *MECP2* genotype. Following gastrostomy, we investigated weight change and carer satisfaction.

## METHODS

**FIGURE 1:**
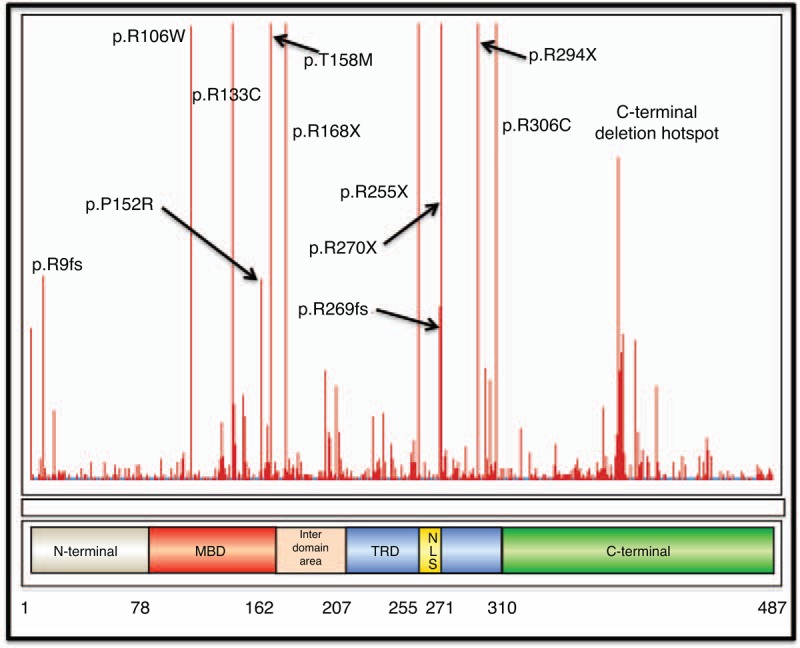
Diagram showing the functional *MECP2* protein and common mutations causing Rett syndrome. Figure derived from RettBASE.^21^ MBD = methyl-binding domain, NLS = nuclear localization sequence, TRD = transcription repression domain. A large deletion includes deletion of all or some of exon 3 and/or 4, and usually includes all or part of the MBD and TRD. An early truncation mutation includes truncation of the protein after aa126, and includes both nonsense and frameshift mutations.

Following registration with the ARSD, a family questionnaire is initially administered and 6 follow-up questionnaires have been distributed between 2000 and 2011. Females with a clinically^18^ or genetically^19,20^ confirmed diagnosis of Rett syndrome and whose parents/carers returned the 2011 follow-up questionnaire were included in this study. Categorization of age and genotype is shown in Table 1. The common mutation types are shown in Figure 1.

**TABLE 1:**
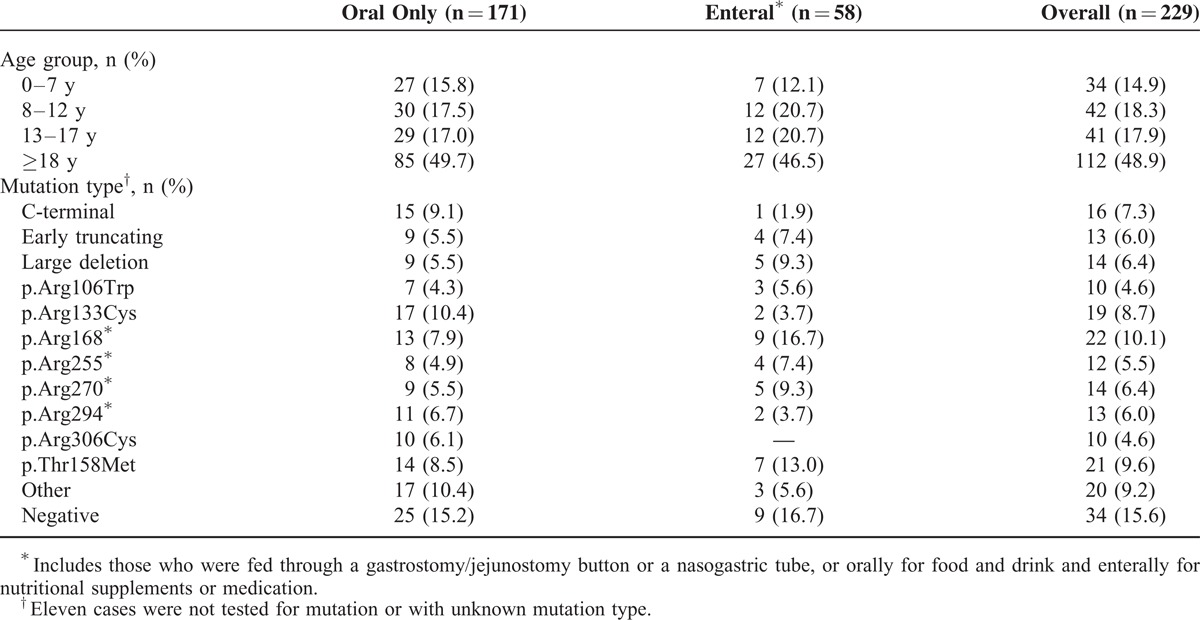
Characteristics of 229 Females Whose Families Responded to the 2011 Follow-Up Questionnaire by Feeding Type

Carers were asked whether the individual was fed orally, enterally, or in combination. If fed orally, caregivers were asked whether the quantity of food eaten was less than, about right, or greater than expected for their daughter's size, their concerns about intake (responses dichotomized by combining “none” or “occasionally” to indicate mild concern and “frequently” or “constantly” to indicate strong concern), and the frequency of coughing, choking, or gagging when eating different types of foods or drinking liquids (classified as absent or present [“less than once per week,” “1–2 times per week,” “daily,” or “more than once per day”]). Average mealtime duration was dichotomized by the median value (20 minutes). For those fed enterally, questionnaire data describing height and weight, dates of gastrostomy insertion, and reason(s) for gastrostomy were supplemented with data from medical records. Weight z-scores for age were calculated using the LMS method based on data from the 2000 US Centers for Disease Control and Prevention Growth dataset.^22^ The reference value of age 20 years was applied to any individual over this age.

Caregivers were also asked about satisfaction or otherwise with the gastrostomy surgical experiences, questions having been developed following evaluation of relevant literature and family input. Items described overall satisfaction, specific aspects of hospital processes, and functional outcomes following surgery, such as weight gain, feeding problem, care, and well-being. Five-point Likert scales were used to rate each question. Families were also asked open questions regarding the aspects of management and outcomes that were satisfactory or otherwise for them.

We interrogated data from earlier questionnaires to investigate relationships between the presence of mealtime issues and subsequent feeding status. For the group who fed orally in 2011, we analyzed responses provided in the 2009 questionnaire, and for those who were later fed via gastrostomy, we used the data provided in the questionnaire prior to gastrostomy insertion.

Ethics approval of the study was obtained from the Human Research Ethics Committee of the Princess Margaret Hospital for Children, Western Australia (1909/EP).

### Statistical Analysis

Logistic regression was used to model the association between *MECP2* mutation type and age group and the binary mealtime issue variables. Generalized estimating equations were used to estimate the occurrence of coughing, choking, and gagging as binary outcome variables for the explanatory variables food type, age group, and mutation type. Logit link function, robust standard errors, and exchangeable working correlation structure were used for parameter estimation. Fisher exact test was used to evaluate equality of proportions. Paired *t* test was used to examine the difference between presurgery and postsurgery weight-for-age z-scores. All missing data were considered missing for reasons unrelated to the study outcomes.

Content analysis was conducted using data from the open-ended satisfaction questions. Data were grouped by the content area of the question and then read and re-read to gain familiarity and form initial ideas of the data set. The researcher then coded recurring words, phrases, or concepts within these areas, and integrated similar codes to define the key themes. The themes were marked within the data to allow further reflection on each thematic decision and either confirm, refute, or modify the original interpretations. A second researcher reviewed all coding decisions to increase credibility of analysis.

All statistical analyses were conducted using STATA release 13 (StataCorp LP, College Station, TX).

## RESULTS

In April 2014, information about 387 females with Rett syndrome born since 1976 had been received from families, among whom 73 had died. Almost a quarter (25.6%, n = 99) had had gastrostomy or jejunostomy feeding tube insertion. The 2011 follow-up questionnaire was administered to caregivers of 262 individuals, including 70 who had gastrostomy surgery, and the response fraction was 87.4% (229/262). The median age of females was 17 years 11 months (range 2 years 8 months to 35 years 9 months). Almost three-quarters (74.7%, 171/229) ate and drank orally including 5 with gastrostomy insertion for other purposes (eg, venting stomach gas), 13.1% (30/229) received their nutrition by a combination of oral and enteral feeds, and 9.6% (22/229) were exclusively enterally fed. Nasogastric tube feeding was rare (2.6%, 6/229). The type of feeding method was similar across age groups (*P* = 0.79). However, oral feeding was more prevalent among those with C-terminal, p.Arg133Cys or p.Arg306Cys mutations, while a higher proportion of those with a p.Arg168^∗^, p.Arg270^∗^, or large deletion^20^ mutations were enterally fed (*P* = 0.13) (Table 1).

### Relationships Between Previous Mealtime Issues and Later Feeding Method

Data on mealtime issues were available in 2009 for the majority (135/166) of females who later fed orally in 2011, and had never been fed enterally. For those who ever had enteral feeding (n = 63), 57% (36/63) of their families provided data on mealtime issues when fed orally as reported in the questionnaire prior to gastrostomy insertion. Close to two fifths with a gastrostomy (36%, 13/36) had previously been perceived as eating less than expected, compared with 10% (13/126) in the oral feeding group (*P* = 0.001) (Table 2). Families of about one-sixth (17%, 3/18) of those with a gastrostomy had had serious concerns about food intake, in contrast to 7% (10/134) of those whose child was fed orally (*P* = 0.19). A larger difference was observed for concerns about fluid intake (gastrostomy: 50% 14/29; oral feeding: 28% 38/134; *P* = 0.05). Coughing during feeding was previously more common among those in the gastrostomy group (68% 21/31) when compared with their orally fed counterparts (12%, 16/135, *P* < 0.001), as was choking (39%, 12/31, *P* < 0.001) and gagging (29%, 9/31, *P* < 0.001) (Table 2).

**TABLE 2:**
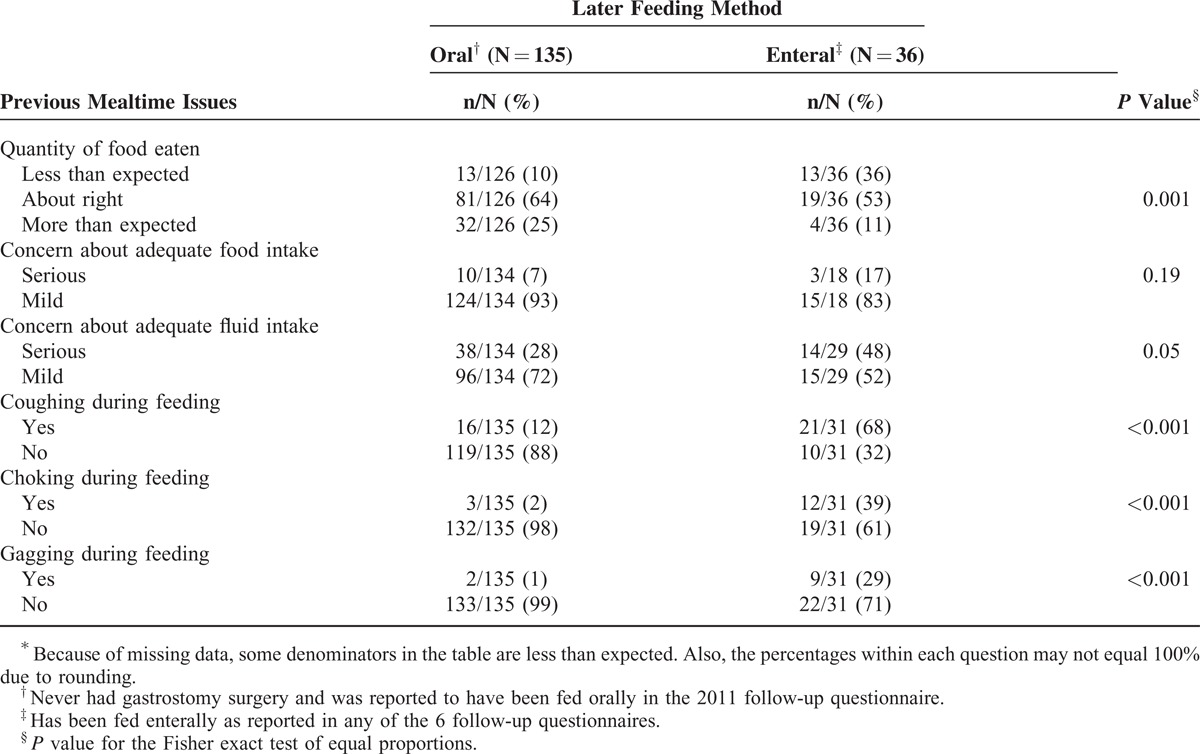
Prevalence of Previous Mealtime Issues and Their Relationship With Later Feeding Method^∗^

### Feeding Difficulties During Oral Feeding

Of the 166 females identified in the 2011 questionnaire as exclusively orally fed and with no history of enteral feeding, slightly more than half (55%, 90/164) of families thought the quantity of food eaten by their child was about right, whereas 15% (25/164) considered the amount was less than expected and 30% (49/164) more than expected. Compared with their adult peers, girls ≤7 years were more likely to eat less than expected than their adult peers (odds ratio [OR] 4.99; 95% confidence interval [CI] 1.26, 19.71), and their families or carers were more likely concerned about food intake (OR 4.50; 95% CI 1.10, 18.35). Adjusted for age, mutation type was not associated with family's perception of food intake (Table 3).

**TABLE 3:**
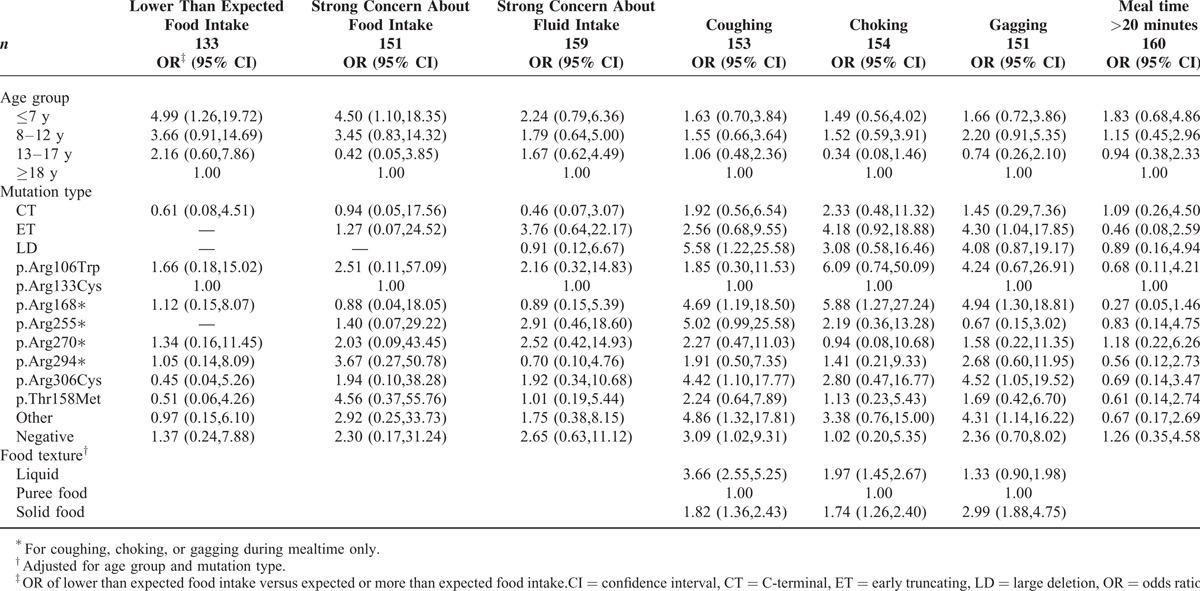
Multivariable Logistic Regression Analysis of Feeding Difficulty Occurrence by Age Group, Mutation Type, and Food Texture^∗^ Among 166 Females Who Were Reported as Orally Fed in the 2011 Family Questionnaire With No History of Gastrostomy

Two-thirds (65%, 108/166) of families reported at least some coughing during feeding and a smaller proportion choking (31%, 51/166) or gagging (37%, 61/166). In 2011, coughing was more likely to be associated with the presence of a large deletion (OR 5.58; 95% CI 1.22, 25.58), p.Arg168^∗^ (OR 4.69; 95% CI 1.19, 18.50), or p.Arg306Cys (OR 4.42; 95% CI 1.10, 17.77) mutations compared to the p.Arg133Cys mutation (Table 3). Using pureed food as the reference category, higher odds of coughing were observed with liquids (OR 3.66; 95% CI 2.55, 5.25) or solid food (OR 1.82; 95% CI 1.36, 2.43) after adjusting for age group and mutation. Compared to those with p.Arg133Cys, females with a p.Arg168^∗^ mutation had higher odds of choking when being fed (OR 5.88; 95% CI 1.27, 27.24). After adjusting for age and mutation, liquid (OR 2.14; 95% CI 1.47, 3.09) and solid (OR 1.69; 95% CI 1.15, 2.47) foods were more likely than pureed foods to be associated with choking. Gagging was more likely to occur in those with p.Arg168^∗^ (OR 4.94; 95% CI 1.30, 18.81), p.Arg306Cys (OR 4.52; 95% CI 1.05, 19.52), and early truncating (OR 4.30; 95% CI 1.04, 17.85) mutations after adjusting for age group. Compared to pureed food, solids (but not liquids) increased the odds of gagging (OR 2.99; 95% CI 1.88, 4.75) (Table 3).

Over half (57%, 94/164) of females stopped eating or drinking at some period, more than half of episodes (54%, 58/108) occurring when unwell. Mealtimes were prolonged (>20 minutes) for just under half (45%, 74/166) of those who were fed orally. Compared to adults, those ≤7 years had slightly increased odds (OR 1.83; 95% CI 0.68, 4.86) of prolonged mealtimes (Table 3).

### Satisfaction With Gastrostomy

The median age at surgery was 8 years 11 months (range 1 year to 35 years and 4 months) and satisfaction data were provided by 63 families at a median period of 6 years 7 months following the procedure. Reasons for gastrostomy/jejunostomy insertion were provided by the carers of 47 females, and included insufficient food and/or fluid intake (48%), feeding difficulties (23%), poor weight gain (13%), temporary illness (9%), and administration of medications (7%). The mean presurgery weight-for-age z-score was −3.50 (n = 42) that increased, on average, by 0.86 (95% CI 0.41, 1.31) at approximately 2 years postsurgery.

In general, carers were satisfied with the surgical procedure and would recommend it to others in similar circumstances (median score 5; interquartile range [IQR] 5,5). They felt less anxious (median score 5; IQR 4,5) and more optimistic in caring for their child (median score 5; IQR 5,5) (Table 4). The majority of carers (>85%) were satisfied with outcomes of reduced feeding difficulties, weight gain, improved general well-being and happiness, and greater ease of daily care. Approximately two thirds were satisfied with outcomes in relation to vomiting, abdominal bloating, and fatigue throughout the day (Table 5).

**TABLE 4:**
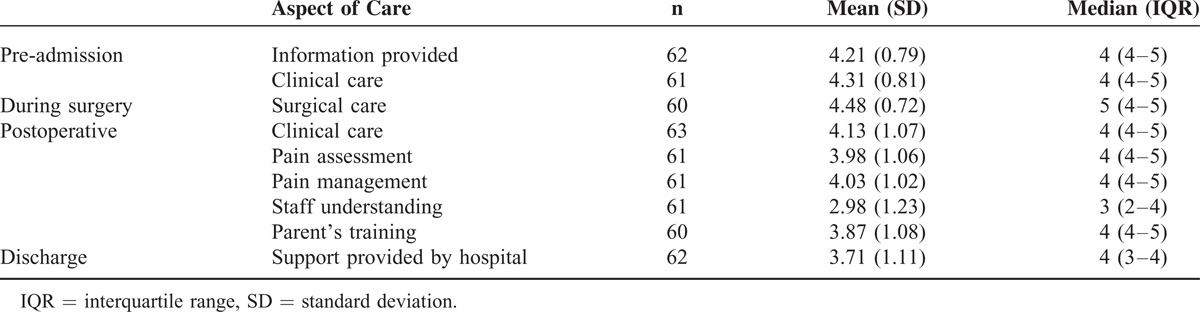
Scores of Parent-Rated Satisfaction With Individual Aspects of Gastrostomy Care

**TABLE 5:**
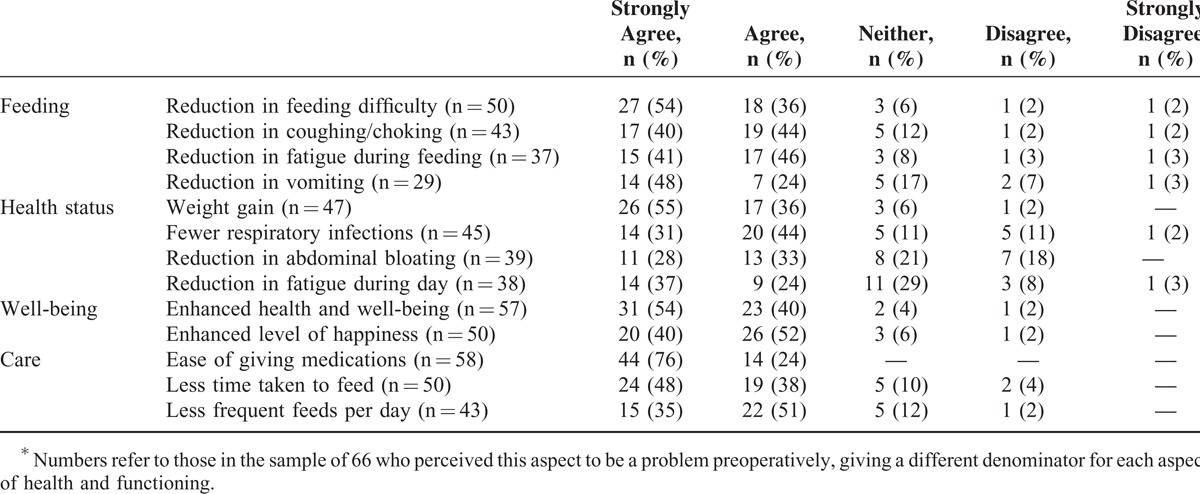
Parent Satisfaction With Changes in Feeding, Health and Well-Being, and Care of Their Daughter Following Gastrostomy Surgery^∗^

Responses to the open-ended questions were provided by the majority of caregivers (92%, 58/63) and themes related to the health of their daughter, family burden and stress, and relationships with health care providers (Table 6). Many described benefits for their daughter's health including weight gain, better respiratory health, and improved energy levels. A small proportion described complications ranging from minor wound problems such as granulation to a more serious adverse effect of catheter migration. One family discussed the compatibility of gastrostomy with continued oral feeding that was pleasurable for their daughter. Many families valued more efficient care routines in relation to feeding and administration of medications via gastrostomy, and reduced worry and stress in relation to mealtime procedures and their daughter's health. Health care providers played important roles in decision-making processes, ongoing management, and education on day-to-day care and routine gastrostomy changes, and relationships that were mutually respectful were highly valued (Table 6).

**TABLE 6:**
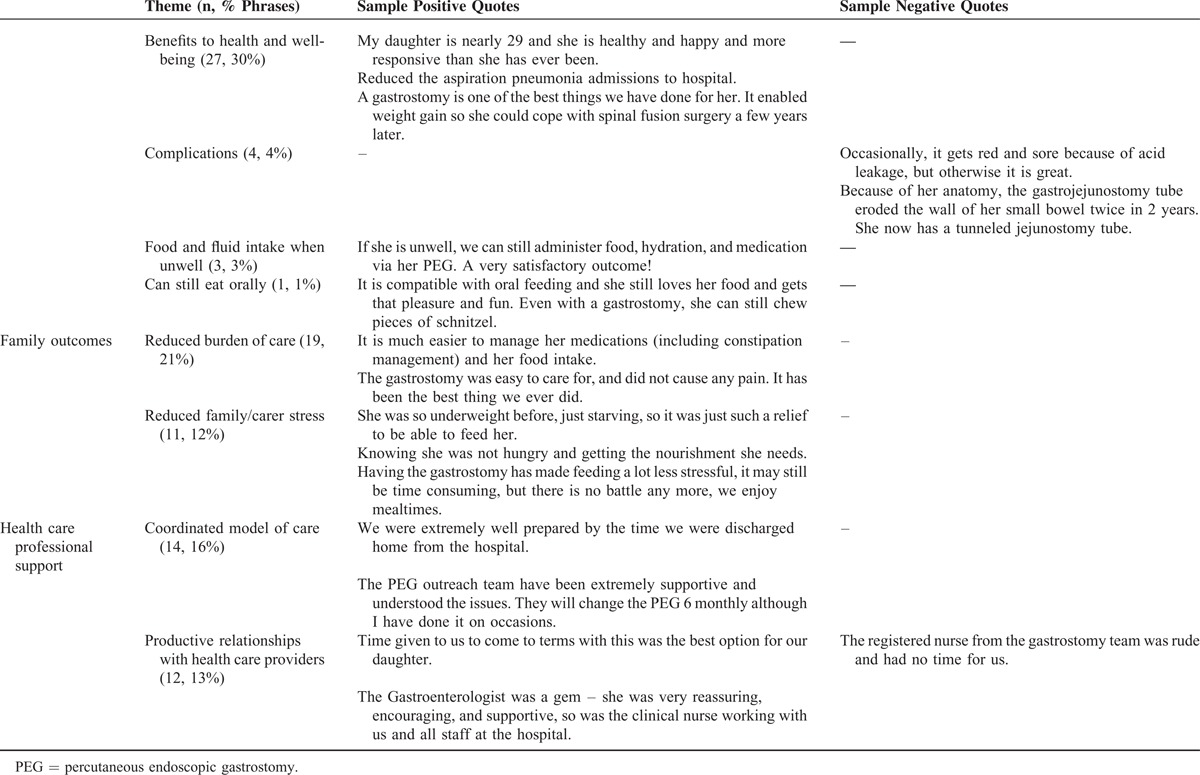
Themes and Sample Quotes Describing Satisfaction Following Gastrostomy

## DISCUSSION

Oral feeding in Rett syndrome is frequently associated with feeding difficulties, including coughing in two thirds and choking and gagging in approximately one third, which occurred more frequently in those with a p.Arg168^∗^ mutation and in those who were later to receive a gastrostomy. Gastrostomy was utilized in approximately one quarter of the Australian Rett syndrome population and consistently resulted in weight gain. Families were satisfied with the gastrostomy procedure and reported improvements in their daughter's health and reduced family stress and burden during daily care activities.

Using longitudinal data in the ARSD, we found that coughing and choking during meals and to a lesser extent *MECP2* genotype predicted the likelihood of gastrostomy. Genotype in Rett syndrome contributes to many aspects of phenotype including clinical severity,^3^ functional abilities,^23^ and comorbidities such as epilepsy.^5^ In Rett syndrome, C-terminal deletions, and p.Arg133Cys and p.Arg306Cys mutations are associated with a milder phenotype and mutations such as large deletions, p.Arg168^∗^ and p.Arg270^∗^ with a more severe phenotype.^3,24–26^ Our study findings were consistent with these observations. For example, those with a mutation usually associated with a milder phenotype, including C-terminal deletions previously found to be associated with better growth,^25^ were more likely to feed orally and less likely to experience feeding difficulties during meals. Conversely, those with the more severe mutations such as p.Arg168^∗^ and large deletions were somewhat more likely to experience feeding difficulties and undergo gastrostomy.

Coughing, choking, and gagging during feeding are likely associated with altered muscle tone and oromotor incoordination^8^ and are important mechanisms to prevent aspiration. Their frequencies in our Rett syndrome cohort suggest that the airways are at risk. Caregiver reports of food texture preferences and tolerance therefore form a part of clinical assessment and may indicate the need for formal assessments such as video fluoroscopy.^10^ Use of pureed foods rather than solid, liquid, or mixed textures was associated with fewer feeding difficulties, and clinical assessment can inform recommendations to modify food texture.^10^ The presence of coughing and choking was nevertheless associated with later gastrostomy. Children who later received a gastrostomy were often perceived previously as eating less than an adequate quantity of food. However, this was not always the case suggesting that clinical discussions regarding growth should extend beyond parent report of adequate food intake. Together with other clinical signs such as the growth trajectory, our findings provide guidance for clinicians when counseling on need and value of gastrostomy.

The decision to consent to gastrostomy is often difficult for families but satisfaction following gastrostomy is high in a range of conditions associated with developmental disability.^12,27^ We also investigated satisfaction in relation to hospital procedures and later child and family outcomes. Strong family satisfaction with hospital procedures extended to education and ongoing coordination of care procedures following discharge, supporting the value of current protocols.^28^ Lowest satisfaction levels related to the lack of understanding of Rett syndrome by ward staff, indicating that general nursing staff need ongoing education to support their management of complex disability. Our Australian experience contrasts favorably with a previously reported qualitative study from the United Kingdom in which families judged that preparation was inadequate in terms of both information and implications for their later lifestyle, and felt excluded from the decision-making processes.^29^

As in a US clinical sample,^15^ we documented weight gain following gastrostomy in our population-based sample. Motil et al^15^ also demonstrated increased height in response to enteral feeding but height was infrequently reported in the clinical records and we were unable to assess this relationship. Together with reduced feeding time and easier administration of medications, this likely contributed to parents feeling less stressed about their daughter's care and her general health and well-being. Air swallowing and subsequent abdominal bloating occurred in approximately 50% of one population-based sample^30^ and gastrostomy has been recommended for venting excess gas from the stomach.^31^ In our study, gastrostomy eased discomfort associated with gas accumulation in the stomach, but if excess gas is located in the small and large bowel, venting via a gastrostomy could be less effective. One family expressed dissatisfaction because their child experienced the complication of catheter migration into the small bowel (a rare complication reported in 2% of 329 children who underwent gastrostomy).^32^ Families were more concerned about their daughter's feeding during the early years when they were also coming to terms with the new challenges of their daughter's diagnosis.

The Australian Rett Syndrome Database is population based and has collected comprehensive data longitudinally since 1993,^17^ giving generalizability to our findings. Importantly, we have identified almost all females with Rett syndrome in Australia who had a gastrostomy since 1993 and administered consistent questions about feeding difficulties, enabling us to examine predictive clinical factors for gastrostomy. Our response fraction for the current study was also high. We were unable to include the whole population in our analysis of the implications of feeding difficulty for later gastrostomy because some were recruited following gastrostomy. Based on review of the literature and family input, we developed a family satisfaction questionnaire in relation to gastrostomy and later child and family outcomes for inclusion in the 2011 follow-up questionnaire. Families were keen to report on their experiences because for many, it had been difficult to accept a gastrostomy and potentially reject the sensory and social benefits of oral feeding for their daughter. We acknowledge that we found little variability in levels of satisfaction and that consistent with Cognitive Dissonance Theory,^33^ parents are more likely to report favorably since they consented to the procedure. As a counterpoint to this limitation, the open-ended questions provided opportunity for both positive and negative aspects. Despite this, the themes we identified were still overwhelmingly positive. Our documented weight gain helped to more fully articulate the clinical outcomes. Clinical outcomes and family satisfaction are both important components of the quality care outcomes framework^16^ and were consistently positive in this study. A proportion of our subjects had not tested positively for a pathogenic *MECP2* mutation. We acknowledge that genetic testing for women with a clinical diagnosis of Rett syndrome has important roles to play, but a proportion of those in our cohort who were older had not been tested for a *MECP2* mutation because of its relative recent availability and some families do not now want to revisit diagnostic processes.

Ongoing clinical care of females with Rett syndrome includes regular monitoring of growth and its influencing factors. Our study identified that coughing, choking, and gagging during meals predict the need for gastrostomy that, in turn, relieves symptoms and promotes weight gain. Clinicians should initiate early discussion with families on the relative merits of gastrostomy and provide emotional support for decision making. Our data may be applicable to a wide range of diagnoses associated with disability, feeding difficulties, and poor weight gain.
